# Dataset of ADCP-derived current profiles and sea level in Buli Bay, East Halmahera, Indonesia

**DOI:** 10.1016/j.dib.2026.113266

**Published:** 2026-09-16

**Authors:** Suliskania Nurfitri, Mohammad Adli Daffa Amrullah, Rima Rachmayani

**Affiliations:** a Research Group of Environmental and Applied Oceanography, Faculty of Earth Sciences and Technology, Institut Teknologi Bandung, Jalan Ganesha 10, 40132 Bandung, Indonesia; b Master Program in Earth Sciences, Faculty of Earth Sciences and Technology, Institut Teknologi Bandung, Jalan Ganesha 10, 40132 Bandung, Indonesia

**Keywords:** Buli Bay, Acoustic Doppler Current Profiler (ADCP), Tidal currents, Hydrodynamic

## Abstract

In this article, we present an *in situ* hydro-oceanographic dataset collected in the semi-enclosed waters of Buli Bay, East Halmahera, Indonesia, from August 29 to September 13, 2022. The observations were acquired using a bottom-mounted, upward-looking 600 kHz Nortek Aquadopp Acoustic Doppler Current Profiler (ADCP) deployed at 0°50′19.50″ N and 128°15′6.41″ E. The original ADCP measurements comprised 2160 current ensembles recorded at 10-minute intervals across multiple vertical bins. The published dataset focuses on the near-surface current at 4.5 m depth, consistent with the objective of characterizing localized near-surface hydrodynamic variability. The 10-minute current observations were averaged to hourly intervals, and the resulting current time series was decomposed into astronomical tidal and non-tidal residual components using least-squares harmonic analysis with the Python UTide package. Synchronized tidal elevation observations are retained at their original 10-minute temporal resolution. The dataset covers one complete spring-neap tidal cycle and provides quantitative information on tidal elevation, near-surface current magnitude and direction, and tidal and residual current variability. The archived dataset is provided in spreadsheet (.xlsx) format and can support coastal hydrodynamic model calibration and validation, as well as studies of tidal circulation and transport processes in complex semi-enclosed and archipelagic coastal environments.

Specifications Table

**Table:** 

Subject	Physical oceanography
Specific subject area	Ocean current and sea level observation
Data format	Filtered near-surface ADCP and sea level data (.xlsx)
Type of data	Processed spreadsheets (.xlsx): These files contain the final quality-controlled dataset, with near-surface hydrodynamic current data at 4.5 m depth reported at uniform hourly intervals. The dataset was produced through harmonic analysis using the Python UTide (Unified Tidal Analysis and Prediction) package and includes resolved astronomical tidal parameters. Sea-level observations are presented separately at 10-minute intervals.
Data collection	Data were acquired using a 600 kHz Nortek Aquadopp Acoustic Doppler Current Profiler (ADCP). The instrument was installed on the seabed in a stationary bottom-mounted protective frame with an upward-looking orientation. Geographic position was determined using a handheld GNSS device and is reported in latitude and longitude referenced to the World Geodetic System 1984 (WGS84). The deployment covered a 15-day measurement period from 29 August to 13 September 2022, producing 2160 ensembles at 10-minute sampling intervals and 1 m vertical bin resolution. Tidal elevation during the observation period was obtained from the instrument's integrated hydrostatic strain-gauge pressure sensor.
Data source location	Institution**:** Bandung Institute of Technology City/Town/Region**:** Buli Bay, East Halmahera, North Maluku Country**:** Indonesia Approximate survey site boundaries (WGS 1984 datum): 1. North: 0°50′ N 2. South: 0°48′30″ N 3. West: 128°14′ E 4. East: 128°16′ E
Stationary Deployment Coordinates	0°50′19.50″ N and 128°15′6.41″ E (data extracted at near-surface depth of 4.5 m)
Data accessibility	The dataset is available at Mendeley data repository with details as follow. **Data identification number (DOI):**10.17.632/4v6hx9n75w.1 **Direct URL to data:**https://www.doi.org/10.17632/4v6hx9n75w.1
Related research article	None

## 1 Value of the Data

• This dataset provides a continuous in situ time series of near-surface current profiles and simultaneous tidal elevation measurements collected in the semi-enclosed waters of Buli Bay, Indonesia, covering one complete spring-neap tidal cycle.
• The decomposition of current velocity into zonal (*u*) and meridional (*v*) components, together with the filtered tidal parameters, provides observations of short-term hydrodynamic variability and tidal current structure within the bay.
• The dataset can support the calibration and validation of three-dimensional hydrodynamic models, particularly for assessing the representation of tidal currents in semi-enclosed and complex archipelagic environments.
• The observed current profiles can also serve as supporting hydrodynamic information for studies of sediment transport, pollutant and oil spill dispersion, and marine debris transport at the local scale. In particular, the current data may be incorporated into particle-tracking models to examine the potential pathways and short-term dispersion of floating or suspended materials within Buli Bay. Broader spatial or temporal applications would require additional and/or more extensive observations.
• The dataset may also provide useful hydrodynamic constraints for studies of ecological connectivity, larval dispersal, and fish spawning, where tidal currents influence the transport and retention of biological particles. However, such applications require biological information, particle release characteristics, and datasets covering relevant spatial and temporal scales beyond those provided here.
• This dataset addresses an important observational gap in the Halmahera Sea region, where bay-scale *in situ* current observations remain limited. The measurements provide a localized reference for evaluating higher-resolution hydrodynamic simulations and for improving understanding of tidal variability in Buli Bay. Nonetheless, because the observations were obtained at a single station over one spring-neap tidal cycle, this dataset is most appropriate for investigating short-term tidal variability and supporting model evaluation at the deployment site, rather than characterizing long-term or bay-wide circulation.
• Users are encouraged to combine this dataset with additional *in situ* measurements, remote-sensing products, or numerical simulations for wider spatial or temporal analyses.

## 2 Background

Buli Bay is a semi-enclosed coastal basin of high socio-economic and industrial importance in eastern Indonesia, located on the eastern margin of Halmahera Island within the Molucca Sea Collision Zone, where the Halmahera and Sangihe subduction systems converge and shape the regional geological setting [1]. The island exhibits marked geomorphological contrasts, with steep volcanic highlands in the west transitioning to eastern lowlands characterized by ophiolite ridges and adjacent coral islands around Buli Bay [2]. Hydrodynamically, Buli Bay is characterized by complex interactions and intricate archipelagic physical features [2]. Structurally, the bay constitutes a shallow shelf system directly linked to the Halmahera Sea through northern channels, while deep-water exchange is constrained by a submarine sill, making the basin highly sensitive to both local tidal forcing and regional circulation processes [[3], [4], [5]]. The Halmahera Sea itself serves as an important eastern pathway for the Indonesian Throughflow (ITF), transporting South Pacific water masses whose variability is influenced by monsoonal forcing, ENSO, and the Halmahera Eddy [[6], [7], [8]].

Regional hydrographic and circulation studies indicate that the Halmahera Sea contains vertically differentiated flow structures, with upper-layer circulation around islands, intermediate southward nontidal flows, and sill overflows that enhance stratification and water mass mixing [[9], [10], [11]]. The area is also characterized by a pronounced thermocline and halocline that generate vertical shear, while seasonal wind reversals and anti-monsoonal rainfall patterns influence sea surface temperature and nearshore circulation in Buli Bay [12]. In semi-enclosed coastal basins, tidal forcing can strongly influence water-level variability, current direction, residual circulation, and exchange processes, with implications for sediment and material transport [13]. However, despite extensive regional studies on the Halmahera Sea and ITF system, *in situ* observations of tidal elevation and near-surface current variability within Buli Bay remain limited. Remote-sensing observations and relatively coarse-resolution ocean models cannot adequately resolve localized tidal currents and short-term flow variability within the bay, highlighting the need for direct observations.

The dataset presented here addresses this observational gap through a continuous *in situ* field deployment in Buli Bay. A bottom-mounted, upward-looking 600 kHz Nortek ADCP was deployed in the western sector of the bay to measure horizontal currents at multiple vertical bins at 10-minute intervals. For the published dataset, the 4.5 m depth bin was selected to characterize near-surface hydrodynamic variability. Synchronized hydrostatic pressure measurements were used to characterize tidal water-level variability. The current observations were subsequently averaged to hourly intervals and decomposed into astronomical tidal and non-tidal residual components using harmonic analysis with UTide. The resulting dataset provides quantitative information on near-surface current magnitude and direction, tidal variability, and the temporal behavior of tidal and residual currents over one complete spring-neap cycle. These observations provide a localized reference for hydrodynamic model calibration and validation and can support further studies of tidal circulation and transport processes in the complex coastal environment of Buli Bay. The geographical characteristics of the deployment site are illustrated in Fig. 1.

**Fig. 1:**
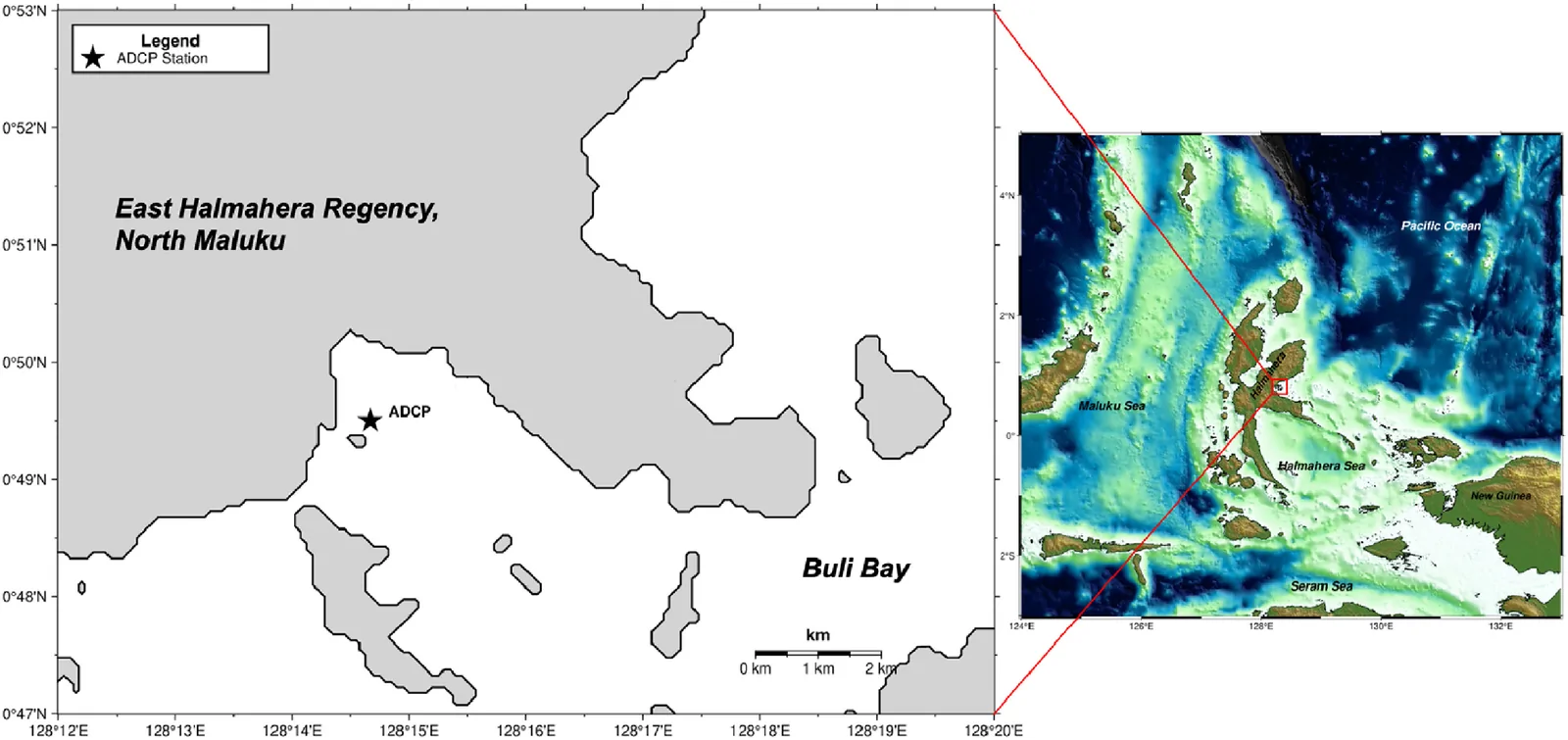
A map of Buli Bay and a station of current measurement using ADCP.

## 3 Data Description

The submitted oceanographic dataset consists of processed current velocity components (*u* and *v*) and tidal elevation data collected in Buli Bay, Indonesia, from 29 August to 13 September 2022. The observations cover approximately 15 days, encompassing one complete spring-neap tidal cycle. The dataset is provided as a spreadsheet file named *current_and_tidal_data_Buli_Bay.xlsx*, which contains two worksheets: current and tides. The *current* worksheet contains the processed horizontal current velocity data, whereas the *tides* worksheet contains the tidal elevation time series.

The original current observations were acquired using an ADCP at 10-minute intervals, resulting in 2160 measurement ensembles during the deployment period. The ADCP measured horizontal current velocity at multiple vertical bins; however, the dataset released here contains the near-surface current measurements at 4.5 m depth. This depth was selected because the primary objective of the dataset is to characterize near-surface hydrodynamic variability in Buli Bay. The current magnitude at this depth provides a representative description of near-surface flow conditions while minimizing contamination from surface and bottom boundary effects. The full vertical current profile is not included, as its characterization is beyond the scope of the present dataset.

The upper four measurement bins, corresponding to approximately the upper 4 m of the water column, were excluded from analysis to minimize potential acoustic sidelobe contamination near the air-sea interface. This exclusion was applied as a quality-control measure appropriate for the bottom-mounted, upward-looking ADCP configuration used in this study. Following this quality-control procedure, the shallowest retained 1-m measurement cell was centered at approximately 4.5 m below the sea surface. This approach ensures the reliability of the near-surface current measurements presented. The deeper vertical bins are therefore not included in the published spreadsheet, as the dataset is specifically tailored to surface-layer processes. For transparency and reproducibility, explicit quality control procedures were applied, including removal of ensembles with invalid pressure readings (< 1 dbar, corresponding to deployment/recovery) and exclusion of velocity outliers based on instrument guidelines. The number of observations removed at each step is detailed in the Experimental Design, Materials, and Methods section.

The original 10-minute current observations at 4.5 m depth were first averaged into hourly values. Each hourly value represents the arithmetic mean of six consecutive 10-minute measurements within the corresponding one-hour interval. Hourly averaging was applied to the current velocity components to reduce short-period variability while retaining the dominant tidal-scale variability. The resulting hourly current time series was subsequently processed using harmonic analysis with the Python package UTide [14] to separate the astronomical tidal and non-tidal residual current components. Thus, the current data provided in the repository represent a processed product derived from the original 10-minute ADCP observations rather than the raw ADCP time series.

In contrast, the tidal elevation observations were retained at their original 10-minute temporal resolution. Therefore, the two data types in the published dataset have intentionally different temporal resolutions: current velocity is provided as hourly averages, whereas tidal elevation is provided at 10-minute intervals. The continuous variation in water-surface elevation recorded by the hydrostatic pressure sensor during the 15-day deployment period is shown in Fig. 2. Retaining the tidal elevation at 10-minute resolution preserves the higher-frequency variability available in the water-level observations.

**Fig. 2:**
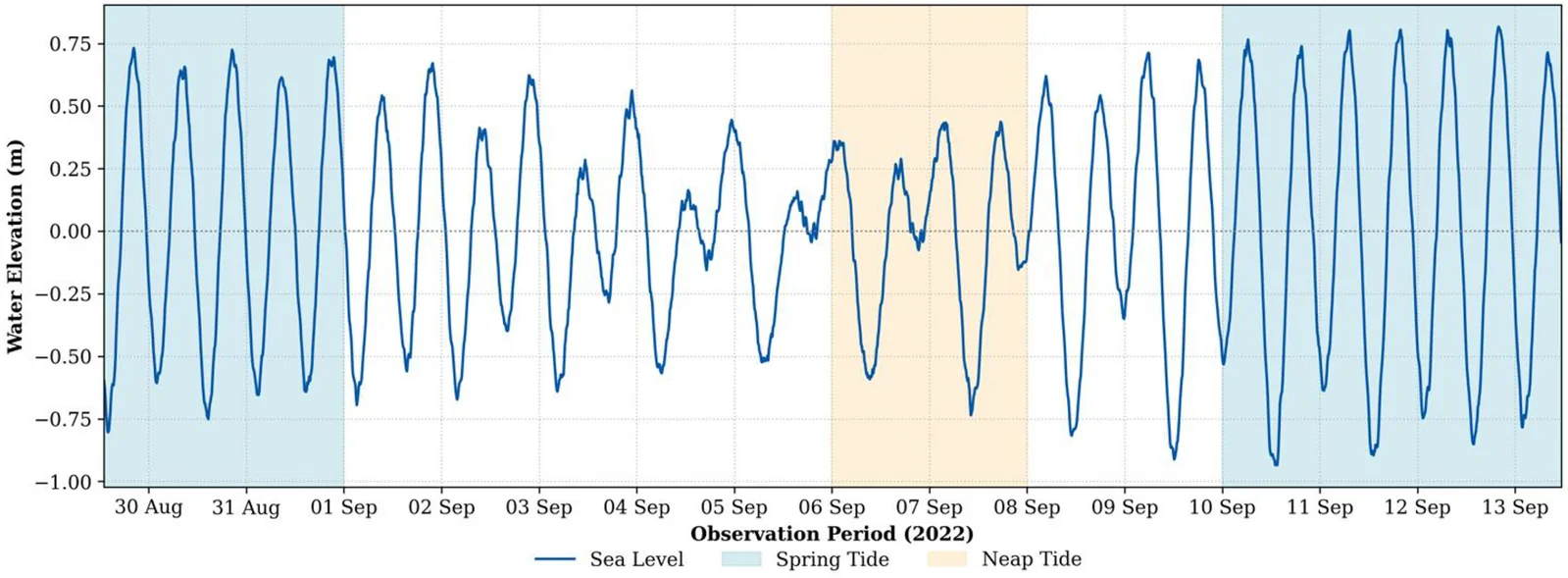
Time series of sea level measured by ADCP relative to MSL.

The *current* worksheet contains the processed hourly current data. The first column, labeled Date & Time, provides the chronological timestamps of the hourly observations. The subsequent columns report the horizontal current velocity components obtained from UTide harmonic analysis. These include Tidal current u and Tidal current v, representing the eastward and northward components of the astronomical tidal current, respectively, and Residual current u and Residual current v, representing the corresponding non-tidal residual current components. All current velocity variables are expressed in m/s.

The *tides* worksheet contains the measured tidal elevation at 10-minute intervals throughout the deployment period. The first column contains the corresponding Date & Time, followed by the tidal elevation values expressed in m. Unlike the current observations, the tidal elevation data were not subjected to hourly averaging and therefore retain the original temporal resolution of the measurements.

Accordingly, the processing and structure of the published dataset can be summarized as follows: (1) the ADCP originally recorded current velocity at multiple vertical bins at 10-minute intervals; (2) the 4.5 m depth was selected to represent the near-surface hydrodynamic conditions relevant to the objective of the dataset; (3) the 10-minute current observations at this depth were averaged into hourly values, with each hourly value calculated from six consecutive measurements; (4) the resulting hourly current time series was analyzed using UTide to separate the astronomical tidal and non-tidal residual current components; and (5) the tidal elevation observations were retained at their original 10-minute resolution. The temporal variability of current magnitude and the separated tidal and residual current components are illustrated in Fig. 3.

**Fig. 3:**
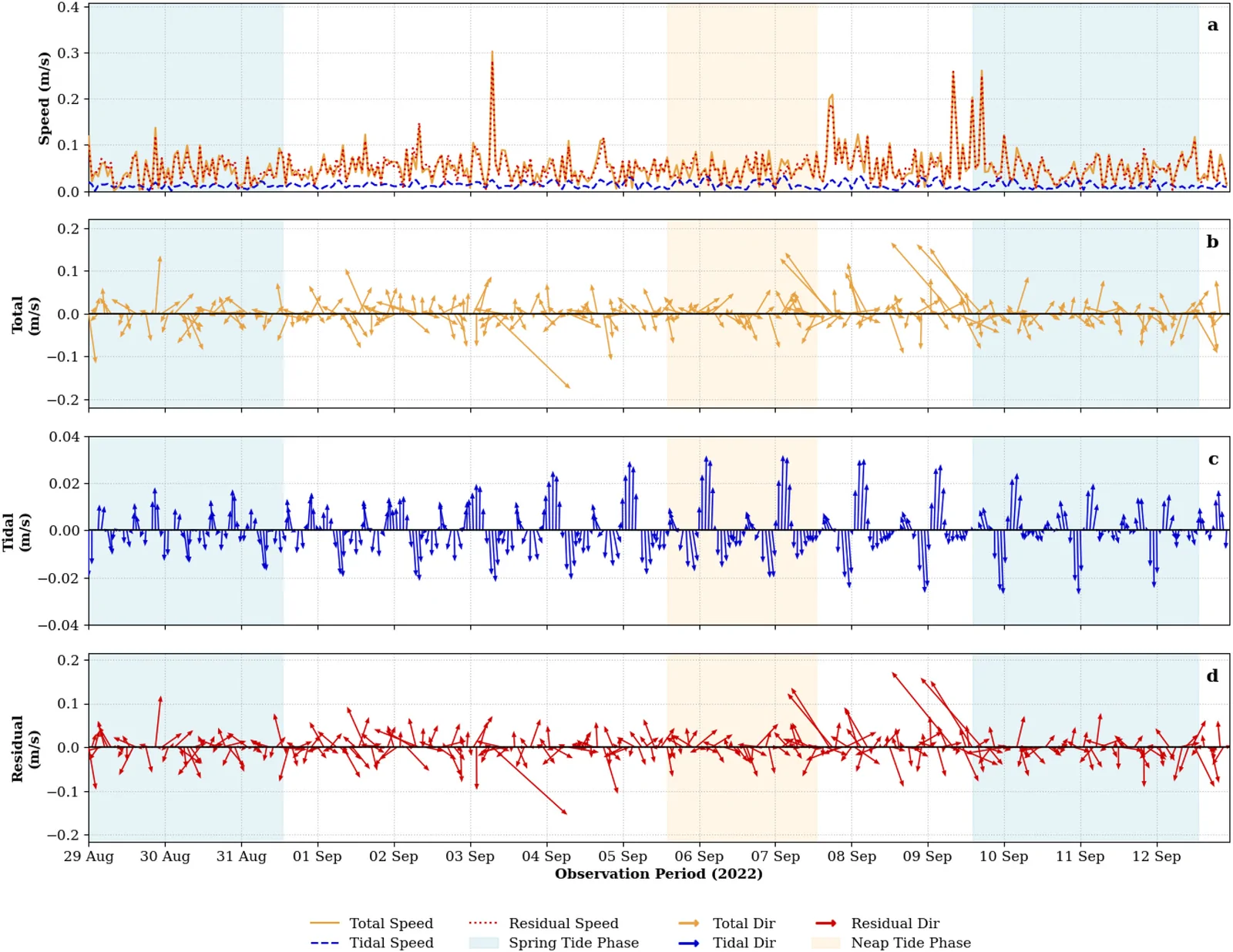
Time series of (a) current magnitude; (b) total current vector; (c) tidal current vector; (d) residual current vector at 4.5 m depth.

The tidal elevation data provided quantitative characteristics of the tidal regime during the observation period. Harmonic analysis identified M2 (0.512 m), S2 (0.288 m), K1 (0.145 m), and O1 (0.114 m) as the dominant tidal constituents, with a Formzahl number of 0.324, indicating a mixed, predominantly semidiurnal tidal regime. The observed water level varied between approximately 1.1 and 2.9 m relative to the local mean sea level (21.148 m), with larger tidal ranges during the spring-tide period and smaller ranges during the neap-tide period. These characteristics provide useful benchmarks for assessing tidal variability and evaluating hydrodynamic model performance in Buli Bay.

The near-surface current data indicate generally low current magnitudes, with speeds typically ranging from 0.02 to 0.10 m/s and occasional peaks of approximately 0.25–0.30 m/s. The tidal current component generally varied within ±0.03 m/s and exhibited a clear bidirectional pattern associated with the flood-ebb cycle, consistent with the predominantly semidiurnal tidal regime. In contrast, the residual current ranged from approximately −0.15 to +0.15 m/s and showed greater directional variability, indicating an appreciable non-tidal contribution to the observed flow. Together, the current magnitude and directional characteristics provide quantitative reference information for near-surface hydrodynamic conditions and model calibration and validation.

For model validation, simulated near-surface horizontal currents at the observation location and corresponding time period can be extracted and compared with the observed *u* and *v* velocity components provided in the *current* worksheet, employing metrics like bias, root-mean-square error (RMSE), correlation coefficient, and discrepancies in current speed and direction. The tidal elevation information in the *tides* worksheet can be autonomously assessed against simulated water levels to verify the depiction of tidal fluctuations. The separation of the measured current into tidal and residual components enables users to evaluate if a model accurately reflects both the periodic tidal signal and the non-tidal flow fluctuations. Given that the dataset pertains to a solitary near-surface station across roughly 15 days, it is best suited for short-term calibration and validation of local hydrodynamic models instead of for constraining seasonal circulation.

## 4 Experimental Design, Materials, and Methods

1 Instrument Installation and Configuration
  The hydro-oceanographic data were acquired using a 600 kHz Nortek ADCP with internal logging capability. The instrument was installed on the seabed of Buli Bay within a stationary bottom-mounted protective frame and configured in an upward-looking orientation to monitor the water column. The blanking distance was set to 0.50 m, and current profiles were recorded at 1.0 m vertical resolution over 25 measurement cells. Measurements were logged continuously at 10-minute intervals. The station position was determined using a handheld GNSS device at 0°50′19.50″ N, 128°15′6.41″ E (WGS84). The field deployment and recovery of the bottom-mounted frame are documented in Fig. 4.

**Fig. 4:**
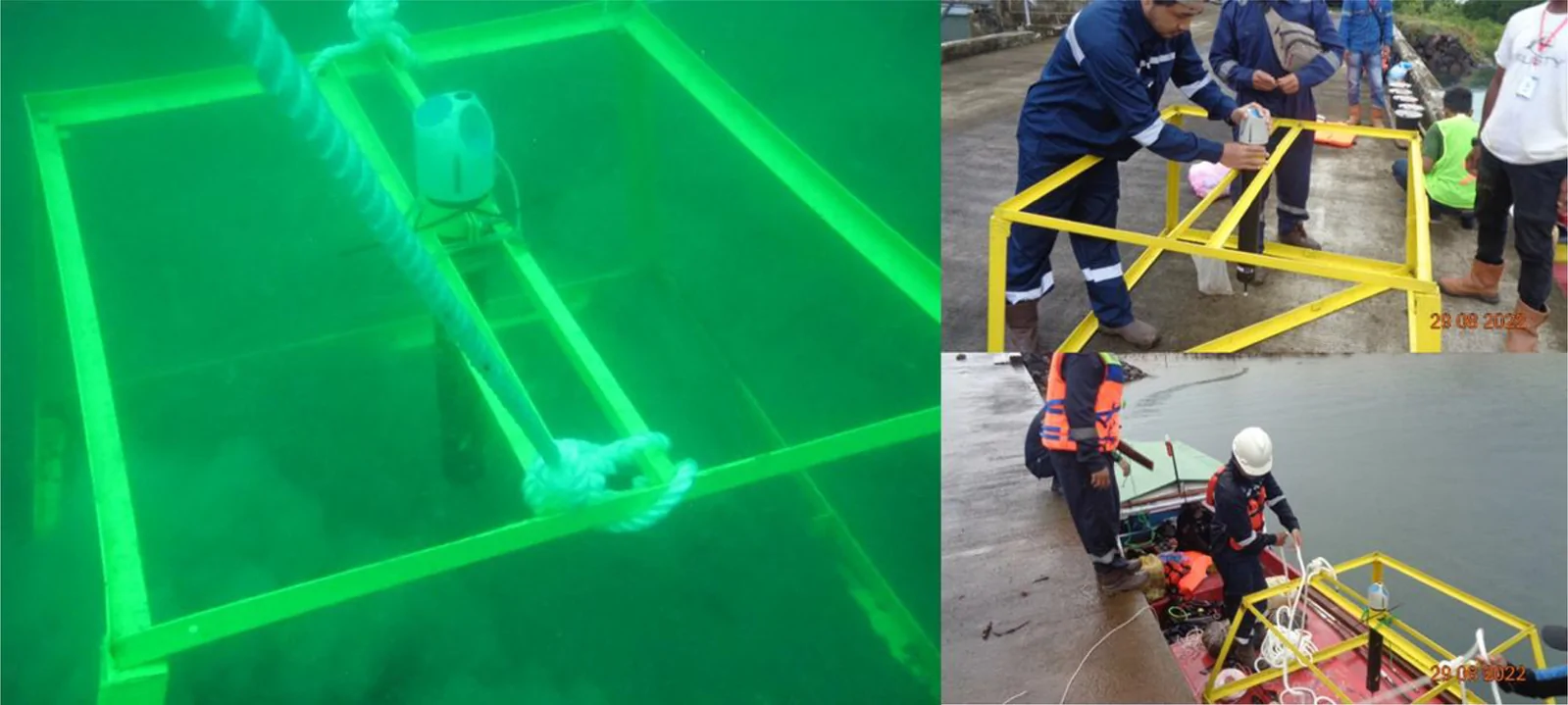
Documentation of ADCP deployment and undeployment.
2 Sequence of Data Acquisition and Processing Pipeline
  Prior to deployment, standard quality assurance procedures were applied, including clock synchronization to Eastern Indonesian Time (UTC+9), compass calibration, and pressure sensor offset correction. Instrument configuration settings were recorded in the native header files (.hdr). The deployment produced 2160 raw measurement ensembles during the 15-day period from 29 August to 13 September 2022, stored in text (.csv) and binary profile (.prf) formats. Data processing was performed using a custom Python script (utide_adcp.py), which imported the raw files and applied quality control to the recorded observations.

Quality control and data-screening steps included:

• Pressure screening: Six ensembles were excluded because the measured pressure was < 1 dbar, corresponding to periods during instrument deployment and recovery.
• Surface sidelobe contamination: The upper four measurement bins were excluded from the velocity analysis because of potential acoustic sidelobe contamination near the air-sea interface.
• Instrument tilt: Instrument orientation remained stable throughout the deployment, with a pitch and roll of 0.8° ± 0.5° and 2.7° ± 0.6°, respectively. All records were within the manufacturer’s recommended threshold of ±5°, so no records were excluded for excessive tilt.
• Velocity outlier detection: Velocity data were visually inspected and statistically screened for outliers. Measurements exceeding ±2 standard deviations from the mean for each time step were flagged and, if present, removed. These parameters were used as quality-control indicators and were not applied as additional corrections to the velocity measurements.

Following quality control, the velocity observations were expressed as eastward (*u*) and northward (*v*) components. A magnetic declination correction of +0.12° E, obtained from the NOAA International Geomagnetic Reference Field (IGRF), was applied to the quality-controlled 10-minute velocity observations before temporal averaging. The corrected *u* and *v* components were then averaged over consecutive one-hour intervals using the arithmetic mean of six observations to reduce high-frequency wave-induced noise [15]. Harmonic analysis was subsequently performed on the hourly *u* and *v* time series to separate the astronomical tidal and non-tidal residual current components. Thus, the processing sequence was (1) raw 10-minute observations; (2) quality control; (3) magnetic declination correction; (4) hourly averaging; (5) UTide harmonic analysis; and (6) tidal and residual current decomposition. The description for each parameter in the final dataset is presented in Table 1.

**Table 1:** Content of the related field dataset (DOI: 10.17632/4v6hx9n75w.1).

Column	Column Header	Parameter Description	Unit
1 2 3 4 5	Date & Time Tidal current *u* Tidal current *v* Residual current *u* Residual current *v*	Timestamps of the field survey Zonal tidal velocity component Meridional tidal velocity component Zonal residual velocity component Meridional residual velocity component	UTC + 09:00 m/s m/s m/s m/s

3. Tidal Decomposition and Peripheral Information

Following hourly averaging, harmonic analysis was applied independently to the *u* and *v* current components using the Python UTide package [14]. The analysis employed the ordinary least-squares method (Eq. (1)), in which the observed velocity at each time step is represented as a superposition of sinusoidal tidal constituents [16].

(1) $$x\left(t_{n}\right)=\bar{x}+\sum_{q=1}^{M}C_{q}\cos\left(2\pi f_{q}t_{n}-\phi_{q}\right)+x_{r}\left(t_{n}\right)$$

In Eq. (1), $\bar{x(t)}$ is the mean value of the record, $x_{r}$ is the residual portion of the time series, $t_{n}=n\Delta t$, and $C_{q}$, $f_{q}$, and $\phi_{q}$ are respectively the constant amplitude, frequency, and phase of the qth constituents [16].

The UTide harmonic analysis used the default setting, fitting the principal semidiurnal and diurnal tides constituents (M2, S2, K1, O1), with 95% confidence intervals computed for each parameter. Tidal predictions were reconstructed from the fitted harmonic constants, and the non-tidal residual *u* and *v* currents were obtained by subtracting the predicted tidal currents from the corresponding observed total currents. The published *current* worksheet contains these processed tidal and residual components. In addition, tidal elevation was derived from the ADCP's integrated hydrostatic pressure sensor. The pressure measurements were converted to water-level elevation (m) and referenced to local mean sea level (MSL), while the original 10-minute temporal resolution was retained.

## Limitations

The 600 kHz ADCP used in this study provided high-resolution current measurements across multiple vertical bins, although its acoustic profiling range was more limited than that of lower-frequency instruments. At the 24-m deep station, the instrument was configured with 1-m measurement cells, 25 bins, and a 0.5-m blanking distance, providing a nominal profiling range of 25 m. The upper portion (four measurement bins) of the profile was excluded from the velocity analysis because of potential acoustic sidelobe contamination near the air-sea interface, an inherent limitation of bottom-mounted upward-looking ADCP measurements. Although the original observations provide information on the vertical current structure, the published dataset is limited to the 4.5 m near-surface current to maintain consistency with the primary objective of characterizing near-surface hydrodynamic variability.

The stationary deployment at a single location also does not capture horizontal spatial variability across Buli Bay. The approximately 15-day observation period covers one complete spring-neap tidal cycle and is therefore suitable for characterizing short-term tidal and near-surface current variability, but it is insufficient to resolve seasonal monsoonal transitions, longer-term circulation changes, and interannual variability. Consequently, applications requiring spatially distributed or longer-term hydrodynamic characterization should combine this dataset with additional *in situ* observations, remote-sensing products, or numerical model simulations.

An additional limitation concerns the reproducibility of the tidal-current decomposition. The published spreadsheet contains the processed tidal and residual *u* and *v* components but does not include the original total current time series. Consequently, users cannot independently repeat the harmonic analysis or test alternative tidal decomposition methods using the released current observations. To support independent verification and further analysis, the original 10-minute current velocity time series at 4.5 m depth is available upon reasonable request to the corresponding author. The documented processing sequence and parameter information provide the basis for interpreting the published components.

## Ethics Statement

The authors have read and followed the ethical requirements for publication in Data in Brief, and this work does not involve human subjects, animal experiments, or any data collected from social media platforms.

## Credit Author Statement

**Suliskania Nurfitri:** Conceptualization, methodology, and writing the original draft; **Moh. Adli Daffa Amrullah:** Data curation and visualization; **Rima Rachmayani:** Writing, review, and editing.

## Declaration of generative AI and AI-assisted technologies in the manuscript preparation process

During the preparation of this work, the authors used Quillbot for grammar checks and refinement and Claude Opus 4.1 for literature synthesis. The authors reviewed and edited the output as needed and take full responsibility for the content of the published article.

## Data Availability

Mendeley DataOcean currents and tide in Buli Bay, Indonesia Dataset (Original data).
